# Effect of Age, High-Fat Diet, and Rat Strain on Serum Biomarkers and Telomere Length and Global DNA Methylation in Peripheral Blood Mononuclear Cells

**DOI:** 10.1038/s41598-018-38192-0

**Published:** 2019-02-13

**Authors:** James M. Antonini, Vamsi Kodali, Terence G. Meighan, Katherine A. Roach, Jenny R. Roberts, Rebecca Salmen, Greg R. Boyce, Patti C. Zeidler-Erdely, Michael Kashon, Aaron Erdely, Mohammad Shoeb

**Affiliations:** Health Effects Laboratory Division, National Institute for Occupational Safety and Health, Morgantown, WV 26505 USA

## Abstract

The objective of the current study was to determine if age, diet, and genetic disposition (animal strain) in an animal model had early effects on specific molecular markers in circulating peripheral blood mononuclear cells (PBMCs). Three strains [Sprague-Dawley (SD), Fischer 344 (F344), and Brown-Norway (BN)] of male rats were maintained on a high-fat (HF) or regular diet. Blood was collected at 4, 12, and 24 wk to assess chemistry and to recover PBMCs. Triglycerides and body weight gain increased at all time points in the HF diet group for each strain. Telomere length in PBMCs decreased in the HF diet group compared to the regular diet group up to 24 wk in all strains. Telomere length decreased in PBMCs at 24 wk compared to baseline in all strains, indicating an age-related effect. These findings highlight that diet and age cause changes in PBMCs recovered from different strains of rats. The next tier of studies will examine the contribution of an occupational exposure (e.g., welding fume inhalation) in combination with diet, age, and strain, to assess changes in the molecular responses of isolated PBMCs. In addition, studies involving lifestyle exposure (e.g., tobacco smoke) are in the planning stages and will assess the long-term effects of exposure in our animal model.

## Introduction

Our long-term study objectives are to^1^: develop a tiered exposure model that will collect longitudinal biological samples during critical ‘occupational’ life stages of an exposed animal that are applicable to human populations and^2^ measure health outcomes to assess multiple factors, such as lifestyle (e.g., diet) and occupational and environmental exposures, which attempt to link a specific internal biological response/endpoint with a specific exposure. An animal model is particularly advantageous for this type of study because of the ability to control all external exposures and to measure potential adverse health outcomes of each animal over its entire lifespan. Also, the genetic contribution to the molecular responses can be assessed using multiple animal strains with varying susceptibilities to unique exposures.

The current report describes the initial study by which three different strains of [Sprague-Dawley (SD), Fischer 344 (F344), and Brown-Norway (BN)] male rats were maintained on a high fat, western (HF) or regular diet for 24 wk. In this first tier of the investigation, the goal was to establish the diet and time course regimen for the different strains of rats. Whole blood was collected at 4, 12, and 24 wk to assess the serum lipid profile and to recover PBMCs for analysis of telomere length ratio and global DNA methylation. The rationale for the choice of the specific strain was based on the need to use strains with varying responses in pulmonary exposure studies that currently are ongoing (tiers 2 and 3). The BN strain has been commonly used in allergic respiratory disease studies due to elevated IgE and Th2 dominant responses. The F344 rat strain also has been extensively used in lung toxicology studies due to susceptibility to pulmonary injury and inflammation, whereas the SD rat is the most widely used outbred strain in animal research, and a large database exists for the SD strain in regards to lung toxicology studies. The information gained from this current preliminary investigation was used in the design and development of ongoing exposure studies examining the added effect of pulmonary inhalation of welding fume and cigarette smoke with diet, age, and rat strain.

## Results

### Body Weight

**Figure 1:**
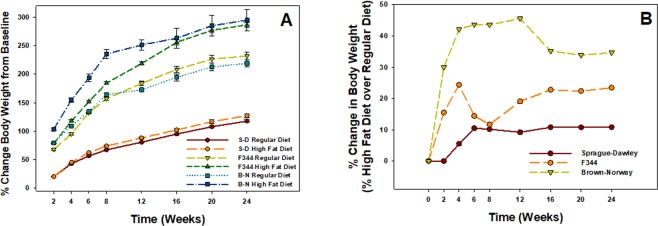
(**A**) Percent change in body weight from corresponding baseline comparing high-fat and regular diets for SD, F344, and BN rat strains over a 24-wk period. Values are means ± standard error (n = 48). (**B**) Percent change in high-fat diet body weight over regular diet body weight for all three strains.

The percent change in body weight over baseline weight was determined for both diet regimens for all three rat strains over the 24-wk period (Fig. 1). In each strain, total body weight (data not shown) and % change in body weight from starting baseline weight increased over time regardless of the type of diet. The % change in body weight above baseline was significantly increased in the high fat diet group compared to regular diet group at every time point for the F344 and BN strains and from 6–24 wk for the SD strain (Fig. 1A). The high-fat diet had the greatest effect on percent change in body weight in the BN strain, causing a 30–40% increase over regular diet from 2–24 wk, whereas the least effect occurred in the SD strain with an approximate increase of 10% from 6–24 wk (Fig. 1B). An intermediate effect of the high-fat diet on the F344 rat strain was observed compared to the other two strains, as a 10–25% increase was observed over the 24-wk period (Fig. 1B).

### Serum Lipid Panel

**Figure 2:**
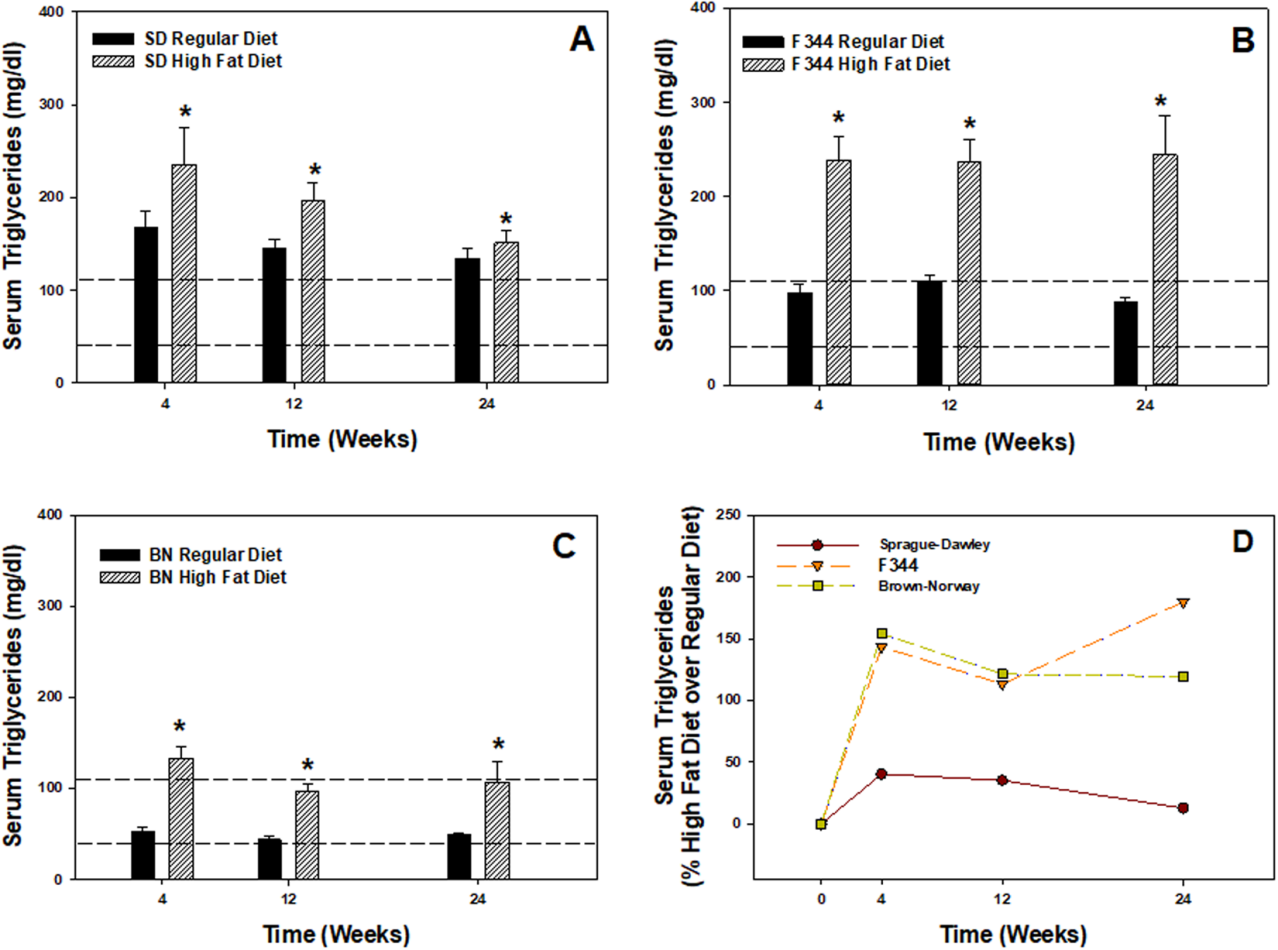
Serum triglyceride values comparing high-fat and regular diets over a 24-wk period for (**A**) SD, (**B**) F344, and (**C**) BN rat strains. Values are means ± standard error (n = 8); *Significantly different from corresponding regular diet group within a time point (p < 0.05). Dashed lines represent normal triglyceride levels (40–110 mg/dl) for the rat. (**D**) Percent change in high-fat diet over regular diet in comparing serum triglyceride values for all three strains.

Lipid analytes were measured in the serum of rats from the different strains at 4, 12, and 24 weeks after maintenance on a high fat or regular diet (Fig. 2, Tables 1 and 2). At all three time points for all three strains, the high-fat diet significantly elevated serum triglycerides compared to the regular diet (Fig. 2A–C). Triglyceride levels were above the range (>110 mg/dl) for what is considered normal at all three time points in the SD strain maintained on the regular diet (Fig. 2A), whereas the levels fell within the normal range for the F344 and BN strains at the three time points when maintained on the regular diet (Fig. 2B,C). In the assessment of age, serum triglyceride levels were not significantly different over the 24-wk time course when comparing the regular diet responses only for each strain (Fig. 2A–C). In regard to percent change in serum triglycerides from high fat diet over regular diet, similar responses to the high fat diet were observed for the F344 and BN strains with approximate increases between 120–180% over regular diet during the 24-wk period (Fig. 2D). The high fat diet had the least effect on serum triglycerides for the SD rats over regular diet with approximate increases of 10–40% during the 24-wk regimen.

**Table 1:** Serum lipid panel comparing high fat and regular diets in three rat strains.

Treatment Group	Total Cholesterol (mg/dl)	HDL (mg/dl)	LDL (mg/dl)
Sprague-Dawley			
Regular Diet-4 wk	66.9 ± 2.70	29.6 ± 0.90	7.50 ± 1.08
High-Fat Diet-4 wk	60.9 ± 4.40	26.4 ± 1.35	8.13 ± 1.16
Regular Diet-12 wk	64.3 ± 2.24	27.3 ± 0.66	6.38 ± 1.33
High-Fat Diet-12 wk	58.8 ± 4.76	25.8 ± 0.66	9.50 ± 0.31
Regular Diet-24 wk	80.3 ± 5.89	27.8 ± 0.93	11.0 ± 0.59
High-Fat Diet-24 wk	84.0 ± 3.27	26.9 ± 0.99	14.0 ± 0.35
F344			
Regular Diet-4 wk	63.3 ± 2.40	27.9 ± 0.77	n.d.
High-Fat Diet-4 wk	64.9 ± 2.85	30.4 ± 0.82	n.d.
Regular Diet-12 wk	71.5 ± 3.18	28.1 ± 0.52	n.d.
High-Fat Diet-12 wk	65.9 ± 3.75	28.9 ± 1.44	n.d.
Regular Diet-24 wk	87.3 ± 5.53	30.4 ± 1.75	n.d.
High-Fat Diet-24 wk	92.0 ± 7.42	35.5 ± 3.26	n.d.
Brown-Norway			
Regular Diet-4 wk	64.3 ± 1.34	23.4 ± 0.25	17.0 ± 0.98
High-Fat Diet-4 wk	72.9 ± 1.47*	26.5 ± 0.73*	20.5 ± 0.73
Regular Diet-12 wk	65.5 ± 1.77	21.1 ± 0.37	15.9 ± 0.78
High-Fat Diet-12 wk	64.5 ± 1.71	22.8 ± 0.42	14.3 ± 0.81
Regular Diet-24 wk	65.5 ± 0.66	26.6 ± 0.50	14.4 ± 0.61
High-Fat Diet-24 wk	70.4 ± 1.37	24.1 ± 0.41	15.1 ± 1.08

Note. n.d. = Below limit of detection; values are means ± standard error (n = 8); *Significantly different from corresponding regular diet group within a time point (p < 0.05).

**Table 2:** Serum lipid panel of a regular diet assessing the effect of age in three rat strains.

Treatment Group	Total Cholesterol (mg/dl)	HDL (mg/dl)	LDL (mg/dl)
Sprague-Dawley			
Regular Diet-4 wk	66.9 ± 2.70	29.6 ± 0.90	7.5 ± 1.08
Regular Diet-12 wk	64.3 ± 2.24	27.3 ± 0.66	6.38 ± 1.33
Regular Diet-24 wk	80.3 ± 5.89*	27.8 ± 0.93	11.0 ± 0.59*
F344			
Regular Diet-4 wk	63.3 ± 2.40	27.9 ± 0.77	n.d.
Regular Diet-12 wk	71.5 ± 3.18^#^	28.1 ± 0.52	n.d.
Regular Diet-24 wk	87.3 ± 5.53^#^	30.4 ± 1.75	n.d.
Brown-Norway			
Regular Diet-4 wk	64.3 ± 1.34	23.4 ± 0.25	17.0 ± 0.98
Regular Diet-12 wk	65.5 ± 1.77	21.1 ± 0.37^	15.9 ± 0.78
Regular Diet-24 wk	70.4 ± 1.37	24.1 ± 0.41	15.1 ± 1.08

Note. n.d. = Below limit of detection; values are means ± standard error (n = 8); *Significantly different from corresponding 4 and 12 wk groups for a specific lipid analyte; ^#^Significantly different from 4 wk group for a specific lipid analyte; ^significantly different from 4 and 24 wk for a specific analyte (p < 0.05).

The effects of diet and age were examined by the measurement of other serum lipid analytes (Tables 1 and 2). A high-fat diet had no effect compared to the regular diet on total cholesterol, HDL, and LDL for the SD and F344 strains at the different time points (Table 1). The high-fat diet caused a significant elevation in total cholesterol and HDL at 4 wk for the BN strain compared to the regular diet (Table 1). When comparing time/age among the groups that were maintained on a regular diet only, total serum cholesterol was significantly elevated at 24 wk for the SD and at 12 and 24 wk F344 strain when assessing only the regular diet as compared to the other time points (Table 2). Serum HDL was significantly increased at 12 wk in the BN strain maintained on the regular diet compared to 4 and 24 wk (Table 2). Serum LDL was significantly decreased only at 24 wk compared to 4 and 12 wk for the SD strain, and not the other two strains, when maintained on the regular diet (Table 2).

### Telomere Length and DNA Methylation Changes

**Figure 3:**
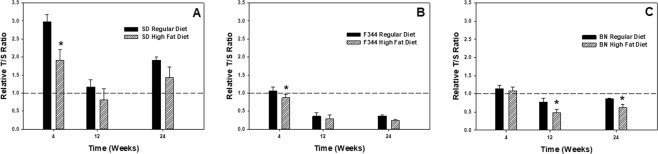
Rat strain effect on telomere length of PBMCs isolated at 4, 12, and 24 wk after maintenance on high-fat (HFD) and regular (RD) diets for (**A**) SD, (**B**) F344, and (**C**) BN strains. Values are means ± standard error (n = 8); *Significantly different from corresponding regular-diet group within a time point (p < 0.05). The relative telomere length was measured by comparing the ratio of telomere repeat copy number (T) and single gene copy number (S) expressed as telomere length (T/S) ratio.

**Figure 4:**
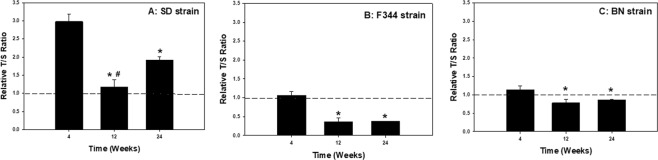
Age effect on telomere length of PBMCs isolated at 4, 12, and 24 wk after maintenance on a regular diet for (**A**) SD, (**B**) F344, and (**C**) BN rat strains. Values are means ± standard error (n = 8); *Significantly different from 4 wk group; ^#^Significantly different from 4 and 24 wk groups (p < 0.05). The relative telomere length was measured by comparing the ratio of telomere repeat copy number (T) and single gene copy number (S) expressed as telomere length (T/S) ratio.

In evaluating the effect of diet on molecular changes, the high-fat diet caused significant shortening of PBMC telomere length at different time points for the different rat strains (Fig. 3). Significant decreases in telomere length were observed for the high-fat diet group compared to the corresponding regular-diet group within a time point at 4 wk for the SD and F344 strains (Fig. 3A,B), and at 12 and 24 wk for the BN strain (Fig. 3C). To highlight the effect of age on these changes in the groups without the influence of diet, PBMC telomere length ratio was compared in the different rat strains at 4, 12, and 24 wk that were maintained on the regular diet only (Fig. 4). For all three strains, the telomere length of isolated PBMCs was significantly decreased at 12 and 24 wk compared to 4 wk. For the F344 and BN strains, PBMC telomere length at 24 wk was not significantly different from 12 wk (Fig. 4B,C), whereas the PBMC telomere length was more variable in the SD strain as the response was significantly different at 12 and 24 wk (Fig. 4A).

**Figure 5:**
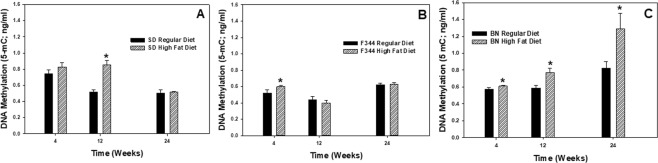
Global DNA methylation as measured by the production of 5-methylcytosine (5-mC) in PBMCs isolated at 4, 12, and 24 wk after maintenance on high-fat (HFD) and regular (RD) diets for (**A**) SD, (**B**) F344, and (**C**) BN strains. Values are means ± standard error (n = 3); *Significantly different from corresponding regular-diet group within a time point (p < 0.05).

**Figure 6:**
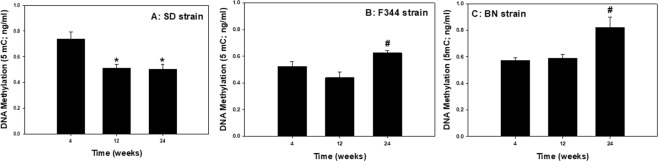
Age effect on global DNA methylation as measured by the production of 5-methylcytosine (5-mC) in PBMCs after maintenance on a regular diet for (**A**) SD, (**B**) F344, and (**C**) BN rat strains. Values are means ± standard error (n = 3); *Significantly different from 4 wk group; ^#^Significantly different from 4 and 12 wk groups (p < 0.05).

To further assess these effect of diet, global DNA methylation of PBMC was evaluated at 4, 12, and 24 wk in the different strains of rats maintained on a regular or high-fat diet. The high-fat diet caused statistically significant increases in PBMC DNA methylation at different time points depending on rat strain (Fig. 5). Significant elevations were observed for the high-fat diet group compared to the corresponding regular diet group within a time point at 12 wk for the SD strain (Fig. 5A), at 4 wk for the F344 strain (Fig. 5B), and all three time points for the BN strain (Fig. 5C). In determining the effect of age on DNA methylation of PBMC independent of diet, DNA methylation of PBMC was compared at 4, 12, and 24 wk in the different strains of rats on the regular diet only (Fig. 6). In the SD strain, PBMC DNA methylation was significantly reduced at 12 and 24 wk compared to 4 wk (Fig. 6A), whereas DNA methylation was significantly increased at 24 wk compared to 4 and 12 wk in the F344 and BN strains (Fig. 6B,C).

**Figure 7:**
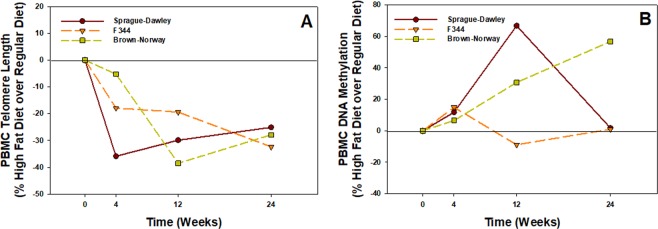
(**A**) Percent change in high-fat diet over regular diet in comparing telomere length for all three strains at 4, 12 and 24 wk. Values are means (n = 8). (**B**) Percent change in high-fat diet over regular diet in comparing DNA methylation of PBMC for all three strains at 4, 12 and 24 wk. Values are means (n = 3).

In a comparison of the three strains, the percent change in high fat over regular diet for PBMC telomere length was the greatest in the SD strain with a decrease of 36% at 4 wk compared to the F344 and BN strains with decreases of 18% and 5%, respectively (Fig. 7A). By 12 wk, the percent change in high-fat over regular diet for PBMC telomere length was less varied for the three strains and nearly the same by 24 wk (Fig. 7A). In comparing the % change in high fat diet over regular diet in regards to DNA methylation of PBMC among the three rat strains, a varied response was observed depending on the strain (Fig. 7B). DNA methylation caused by the high fat diet increased progressively over the time course for the BN strain, reaching a maximum increase of 57% over regular diet at 24 wk, whereas the response of the SD strain to the high-fat diet peaked at 12 wk (67%) and was not different from the regular diet value (2%) by 24 wk (Fig. 7B). The high fat diet had the least effect on DNA methylation compared to regular diet in the F344 strain over the 24-wk period (Fig. 7B).

## Discussion

The long-term goal of this investigation was to establish an animal model to assess the effects of age, strain, and diet on different pulmonary exposures. An animal model has advantages over using human populations as it is nearly impossible to relate health outcomes to the numerous daily influence from the environment, work, and diet of a human throughout different stages of life from conception to death. With an animal model, most exposures for each animal can be controlled over its entire lifespan. In addition, the use of multiple animal strains with differing susceptibilities to various exposures will allow for an assessment of the genetic contribution to the responses measured. The objective of the current study was to determine if age, diet, and genetic disposition (animal strain) in an animal model had early effects on specific molecular markers in circulating PBMCs that may be predictive for health effects later in life. This is the first study that we are aware of that has compared these effects of a high-fat diet at different time points up to 24 wk in outbred and inbred rat strains.

The high-fat diet that was selected in the current study is well-established and has been used to mimic the dietary habits of an obese human and produce adverse health disorders, such as obesity, insulin resistance, and cardiovascular disease^1,2^. The high fat diet, often referred to as the “western diet” or “cafeteria diet”, contained 45% of calories from fat, 36% calories from carbohydrates, and 19% from protein compared to a regular rat diet that consisted of a caloric composition of 18% from fat, 58% from carbohydrates, and 24% from protein. Animals from all three strains were started on the high fat diet at 6 wk of age, which is representative of adolescence for rats. The justification for using a young rat was to model unhealthy eating habits at an early age. One outbred (SD) and two inbred (F344 and BN) strains were used. One limitation of the study was the use of only one sex. Because of the complexity of the investigation (multiple strains, diets, time points, and eventually environmental exposures), we decided to examine the responses in males first. From the results of the current study in male rats, we will be able to better fine tune the design of the study in the females and hopefully, reduce the number of rats needed to complete the study.

To ensure that diet induced physiological effects, body weight changes and a serum lipid panel were evaluated in the high-fat diet group and compared to those animals maintained on the regular diet. As expected, the high fat diet caused a significant increase in % change in body weight above baseline compared to regular diet group throughout the time 24-wk period for all three strains. In comparing the percent change in high-fat diet over regular diet body weight, a varied response was observed depending on the strain examined. The high-fat diet had the greatest effect in regards to percent body weight change in the two naturally more lean, inbred strains (F344 and BN) compared to the SD outbred strain. Serum triglycerides also were significantly elevated in the three strains at all three time points examined in the high-fat diet group compared to the regular diet group. Variations in the absolute serum triglyceride values were observed in the responses for the different strains. Similar responses with an enhanced effect in serum triglyceride values were observed for the two inbred strains after maintenance on the high-fat diet compared to the SD outbred strain. The high-fat diet had little to no effect on serum cholesterol, LDL, and HDL in the three strains over the 24-wk time course.

One possible mechanism by which dietary changes may influence transcriptional regulation is through a persistent alteration in gene regulation that does not involve changes to the DNA sequence^3^. A commonly studied molecular modification is the measurement of telomere length. Telomeres are nucleoprotein structures composed of simple repetitive DNA sequences (TTAGGG)_n_ that stabilize ends of chromosomes by preserving genetic information and preventing DNA degradation^4^. Environmental factors affect telomere length throughout one’s lifespan^5^. Telomeres shorten with age, and their length has been shown to be influenced by diet, chronic inflammation, physical activity, and environmental and occupational exposures^6–8^. Importantly, changes in telomere length have been associated with adverse health effects, such as an increased risk of cancer development^9,10^.

Numerous studies have examined the effect of diet on leukocyte telomere length in human subjects (as reviewed by)^11–13^. Beneficial health effects were observed with the intake of fruits and vegetables^14,15^ and adherence to the Mediterranean diet^16,17^, as evidence by longer telomere lengths, whereas the intake of diet containing high amounts of processed meats^18,19^, fats/oils^14,20^, and sugar-sweetened drinks^21^ have been associated with shorter telomere lengths. However, more epidemiological studies are needed to fill the knowledge gaps when evaluating dietary effects on telomere length^11,12^. The potential relationship between telomere length and diet using animal models is lacking. In the current study, the high-fat diet caused significant shortening in telomere length of isolated PBMCs at different time points during the 24-wk time period, depending on strain. Importantly, telomere length of the isolated PBMCs for all three rat strains shortened over time with age (independent of diet) as it was significantly decreased at 12 and 24 wk compared to 4 wk. Of the three strains, the outbred SD strain had the most variable telomere length response in regards to diet and age compared to the two inbred strains.

Another commonly examined molecular and genetic modification is DNA methylation- a covalent addition of a methyl group to cytosine in the context of a cytosine-guanine dinucleotide. DNA methylation can modulate gene expression and has been shown to vary in response to different environmental stressors^22^. It has been observed that prolonged high-fat diet maintenance during adulthood in mice dynamically changed DNA methylation and expression of genes in fat-specific depots, such as gonadal adipose tissue^23^. In addition, a high-fat diet resulted in hyper-methylation and decreased transcription and expression of important hepatic genes controlling lipid homeostasis in a mouse model^24^. The influence of diet on global DNA methylation of PBMCs in the current study was more varied compared to telomere length, but also dependent on animal strain. The high-fat diet had the greatest effect on DNA hyper-methylation for the BN and SD strain but not the F344 strain over the 24-wk time course. When examining the effect of age on DNA methylation without the influence of diet, the response was even more varied depending on strain. DNA methylation of PBMC was reduced at 12 and 24 wk in the SD strain, but increased at 24 wk in the F344 and BN strains.

In this phase of our investigation, it was demonstrated that diet, age, and animal strain induced early changes in molecular responses in isolated PBMCs. Because of the ease of isolation from collected whole blood samples, PBMCs may serve as an important cell type in the study of genetic changes in different populations or occupational cohorts to examine past environmental and occupational exposure as well as predict future progression and development of disease. Importantly, both longer^10,25^ and shorter^9,26^ telomeres in peripheral blood have been associated with an elevated risk of different cancer types. The next tier of studies are currently examining the contribution of an occupational exposure, welding fume inhalation, in combination with diet, age, and rat strain differences on the molecular responses in isolated PBMCs. In addition, studies involving environmental or lifestyle exposure (e.g., tobacco smoke) are in the planning stages and will assess the long-term effects of exposure in our animal model.

## Materials and Methods

### Animals and Diet

Male Sprague-Dawley [Hla: (SD) CVF; Hilltop Lab Animals, Scottdale, PA], Fischer-344 (F344/NHla CVF; Hilltop Lab Animals), and Brown-Norway (BN/RijHsd; Harlan Laboratories, Inc., Indianapolis, IN) rats were received at 5 wk of age and were free of viral pathogens, parasites, mycoplasmas, *Helicobacter*, and *CAR Bacillus*. The rats from each strain (n = 112/rat strain) were acclimated for 5 days after arrival and were provided tap water and irradiated Teklad 2918 standard 18% protein rodent diet (Envigo Teklad Diets, Madison, WI) *ad libitum*. A possible limitation of the study may have been the acclimation period of less that at least a week as shipping can introduce stress, changes in eating habits, and hormonal imbalances^27–29^. Selected nutritional composition of the Teklad 2918 standard diet was 18.6% protein, 44.2% carbohydrate, and 6.2% fat.

After acclimation, sets of animals from each strain were continued on the standard Teklad 2918 diet (n = 48 rats/strain) or maintained on the Teklad Custom 45% Fat Kcal western diet (Envigo Teklad; n = 48/strain) *ad libitum*. The 45% Fat Kcal diet was designed with similarities to the western Diet with the addition of 21% anhydrous milk fat and 34% sucrose. Soybean (2%) was included in the high fat diet to supplement essential fatty acids. Selected nutritional composition of the high fat, western diet was 14.8% protein, 40.6% carbohydrate, and 44.6% fat. Animals were maintained on the standard or high fat, western diets until sacrifice at 4, 12, and 24 wk. All animal procedures used during the study were reviewed and approved by the CDC-Morgantown Animal Care and Use Committee. The animal facilities are specific pathogen-free, environmentally controlled, and accredited by the AAALAC. All methods were performed in accordance with the relevant guidelines and regulations by CDC-NIOSH and AAALAC.

### Serum Collection and Isolation of PBMCs

At 0 (baseline), 4, 12, and 24 wk, serum from whole blood was collected via the abdominal vena cava using an 18-gauge needle and delivered to BD Vacutainer tubes (Becton, Dickinson, and Co., Franklin Lakes, NJ). A Lipid Profile Rodent Panel (total cholesterol, triglycerides, high-density lipoprotein (HDL) cholesterol, and low-density lipoprotein (LDL) cholesterol) was performed on the collected serum using the IDEXX BioResearch platform (North Grafton, MA).

PBMCs were isolated as previously described by Erdely *et al*.^30^ and Shoeb *et al*.^8^ from heparinized blood collected from each animal using Accuspin 12-ml tubes (Sigma-Aldrich Co., St. Louis, MO) containing Histopaque-1083 (Sigma-Aldrich Co.) and centrifuging at 1250 x g for 30 min. PBMCs were collected in sterile 15-ml plastic tubes and washed once with PBS at 450 x g for 20 min. Finally, PBMCs were re-suspended in 1 ml PBS (Lonza, Walkersville, MD) for cell counting and genomic DNA (gDNA) isolation. All steps were performed at room temperature.

### DNA Isolation and Telomere Quantitative PCR (qPCR) Assay

gDNA was extracted from the PBMCs isolated from whole blood using DNeasy Blood & Tissue Kit (Qiagen Sciences Inc., Germantown, MD). DNA concentration was measured using the Nano-Drop 2000 spectrophotometer. Samples were diluted to a final concentration of 25 ng/1.5 μl to measure telomere length as previously described by Shoeb *et al*.^8^. Quantitative PCR was performed using the SYBR Select Master Mix (Life Technologies, Carlsbad, CA) with a step one plus real time PCR system (Applied Biosystems, Foster City, CA). The parameters used were as follows: 95 °C for 10 min (enzyme activation), 95 °C for 15 sec (denaturing), and 60 °C for 60 sec (annealing), 60 cycles. Primers used were as follows: Tel rat-F 5′-GGT TTT TGA GGG TGA GGG TGA GGG TGA GGG TGA GGG t-3′, Tel rat-R 5′-TCC CGA CTA TCC CTA TCC CTA TCC CTA TCC CTA TCC CTA- 3′; AT1 rat-F 5′-ACG TGT TCT CAG CAT CGA CCG CTA CC-3′, and AT1 rat-R 5′-AGA ATG ATA AGG AAA GGG AAC AAG AAG CCC-3′ (Invitrogen Corporation, Carlsbad, CA). The relative telomere length was measured by comparing the ratio of telomere repeat copy number (T as Tel1) and single gene copy number (S as AT1), expressed as telomere length (T/S) ratio. Each individual values obtained by qPCR were processed through the formula T/S = 2^−ΔCT^, where ΔCT = CT_telomere_ − CT_AT1_. This ratio was then compared with the ratio of the reference DNA. Each DNA sample collected was measured in duplicate.

### DNA Methylation

DNA Colorimetric Quantification Kit (Abcam, Cambridge, UK) was used to determine global DNA methylation of isolated PBMCs according to manufacturer instructions. Briefly, binding buffer was added to each well then a negative control, positive control, or 400 ng of gDNA per reaction was added. The plate was incubated for 90 min, washed, and incubated with capture antibody for 60 min. Then, washed and incubated with detection antibody after which an enhancer solution was added. Finally, the plate was washed and developing solution was added followed by stop solution. Absorbance was read at 450 nm. DNA Methylation was determined as measured by the production of 5-methylcytosine (5-mC).

### Statistical Analysis

Statistical analyses were performed using JMP version 12 and SAS version 9.4 for Windows (SAS Institute, Cary NC). Factorial analysis of variance (ANOVA) was performed with a Tukey-Kramer HSD post-hoc test to make pairwise comparisons for each variable among all treatment groups for a strain across time. For some variables (serum triglyceride and HDL), data were log transformed to reduce heterogeneous variance and meet the assumptions of an ANOVA. For all analyses, a p value of <0.05 was set as the criterion for significance.

## Data Availability

The datasets generated during and/or analyzed during the current study are available upon request from the corresponding author on reasonable request.
